# Supplementary material for: Confidence and knowledge in emergency management among medical students across Colombia: A role for the WHO basic emergency care course
Source: PLoS One. 2022 Jan 21;17(1):e0262282. doi: 10.1371/journal.pone.0262282 (PMC8782416; doi:10.1371/journal.pone.0262282)
Supplement: S1 File — (PDF) [file pone.0262282.s001.pdf]

Consentimiento

Por favor, complete la encuesta a continuacin.□

Gracias!

Permiso Para Tomar Parte en un Estudio de Investigación:

Información Clave

Lo siguiente es una breve descripción de la investigación en que le estamos invitando a participar. Pueden decidir si quieren participar o no.
Más información estará detallada a continuación.

¿Por qué me han invitado a participar en este estudio?

Se le ha invitado a participar en este estudio porque usted esta en su último año de escuela de medicina en Colombia. Cual nos indica que va a cumplir su año de servicio social el año que viene.

¿Qué es lo que debo de saber sobre este estudio?

- Usted decide si quiere participar o no
- Sus respuestas son anónimas y no se compartirán con su escuela de medicina
- Puede elegir no participar
- Puede aceptar participar y luego cambiar de opinión
- Su decisión no tendrá consecuencias en su contra
- Puede hacer todas las preguntas que quiera, antes de decidir

¿Por qué se esta haciendo esta investigación?

Como estudiantes de último año, ustedes serán los primeros que se van a enfrentar con urgencias en la clínicas de Colombia. Esta investigación ayudara lanzar el curso de la Organización Mundial de la Salud diseñado para enseñar las habilidades practicas relacionados con atención a urgencias medicas con la meta de mejorar tu cuido de pacientes durante el próximo año.

¿Cuánto tiempo tomara ser parte de esta investigación?

Se calcula que su participación tomara 12 meses. Durante este tiempo, tendrá que participar en 3 encuestas en línea.

¿Es posible que mi participación pudiera afectarme negativamente?

Sus respuestas a las preguntas serán completamente anónimas. La evaluación de su conocimiento no afectara su calificación en la escuela o su colocación de empleo. Sus respuestas no serán compartidas con su escuela de medicina.

¿Qué pasa si no quiero ser parte de esta investigación?

Su participación es completamente voluntaria. Puede decidir si quiere participar o no. No tendrá consecuencias en su contra si decide no participar.

Información Detallada

Sobre este formulario

Favor de leer este formulario cuidadosamente. Contiene información sobre lo que significa su participación en esta investigación. Tiene el derecho de tomar su tiempo en lo que decide si quiere participar. Si tiene alguna pregunta o duda sobre la investigación o sobre este formulario puede preguntar en cualquier momento. Si decide participar, se le pedirá que firme este formulario. Se le dará una copia.

Consentimiento

Permiso Para Tomar Parte en un Estudio de Investigación:

¿A quien se le hace preguntas?
Si tiene alguna pregunta, duda o queja favor de hablar con el equipo de investigación. Correo electrónico: katelyn_moretti@brown.edu.

Participación es voluntaria
A sido invitado a participar por ser estudiante de medicina, en su último año en Colombia. Usted elige si quiere participar o no. Si elige participar, puede cambiar de opinión y retirarse de la investigación en cualquier momento. Negarse a participar no tendrá ningún efecto negativo o pérdida de los beneficios que le corresponden.

¿Cuántas personas participaran en la investigación?
4,000 personas serán invitadas a participar en esta investigación.

¿Qué puedo esperar si participo en el estudio?
Al ser un participante, hay la expectativa de completar 3 encuestas sobre unos 12 meses. La encuestas son de 25 preguntas, con el fin de evaluar su comodidad en el manejo de urgencias medicas y su conocimiento sobre los temas esenciales del cuido de urgencias.

¿Cuáles serán mis responsabilidades?
Como participante, usted será responsable por completar 3 encuestas de 25 preguntas sobre el transcurso de los próximos 12 meses. Tiene la opción de participar en una entrevista sobre que piensa usted que son los desafíos enfrentados en el cuido urgente de poblaciones de pacientes marginados

¿Cuáles son los beneficios de participar en esta investigación?
Su participación ayudara brindar mejor conocimiento sobre las necesidades y los vacíos existentes en el cuido de urgencias en Colombia.

¿Qué pasa si digo que si, y después cambio de opinión?
Se puede retirar de la investigación. No decisión no tendrá ningún reproche.

¿Seré compensado por participar en la investigación?
No recibirá compensación por participar.

¿Como van a proteger mi privacidad, si decido participar? ¿Que hacen con la información?
A usted se le asignará un numero de identificación personal, que estará vinculada con una base de datos. Durante el análisis toda información que pudiera ser utilizada para identificarle, estará destruida.

Declaración de consentimiento
He leído toda la información en este formulario sobre los riesgos y posibles beneficios. Afirmo que todas mis preguntas sobre la investigación han sido respondidas satisfactoriamente. Entiendo que estoy libre a retirarme en cualquier momento sin efecto negativo o pérdida de los beneficios que me corresponden.

Al firmar a continuación, usted da su consentimiento para participar en la siguiente encuesta.

Al participar en este estudio, usted participará en 3 encuestas durante 6 meses. Cada encuesta durará entre 15 y 30 minutos.

¿Dará su consentimiento participar en este estudio longitudinal?

◯ Sí
◯ No

Para futuros contactos, ¿cuál es su email preferido?

____

"Doy permiso para participar en la siguiente investigación de encuesta".

____

Demografía

¿Cuál es su género?

☐ Masculino
☐ Femenino
☐ Otro

¿Cuántos años tiene?

____

¿Cuál es su nacionalidad?

◯ Colombiano
◯ Venezolano
◯ Otra

¿Cuál es su nacionalidad?

____

¿En cuál universidad está estudiando?

- ◯ Católica del Norte Fundación Universitaria
- ◯ Corporación Universitaria Empresarial Alexander von Humboldt
- ◯ Corporación Universitaria Rafael Núñez
- ◯ Corporación Universitaria Remington (Medellín)
- ◯ Fundación Universitaria Autónoma de las Américas (Pereira)
- ◯ Fundación Universitaria de Ciencias de la Salud
- ◯ Fundación Universitaria Navarra
- ◯ Fundación Universitaria Sanitas
- ◯ Pontificia Universidad Javeriana (Bogotá)
- ◯ Pontificia Universidad Javeriana (Cali)
- ◯ Unidad Central del Valle del Cauca
- ◯ Universidad Antonio Nariño
- ◯ Universidad Autónoma de Bucaramanga
- ◯ Universidad CES
- ◯ Universidad Cooperativa de Colombia (Medellín)
- ◯ Universidad Cooperativa de Colombia (Santa Marta)
- ◯ Universidad Cooperativa de Colombia (Villavicencio)
- ◯ Universidad Cooperativa de Colombia en Pasto
- ◯ Universidad de Antioquia
- ◯ Universidad de Boyacá
- ◯ Universidad de Caldas
- ◯ Universidad de Cartagena
- ◯ Universidad de Ciencias Aplicadas y Ambientales
- ◯ Universidad de La Sabana
- ◯ Universidad de los Andes
- ◯ Universidad de Manizales
- ◯ Universidad de Nariño
- ◯ Universidad de Santander
- ◯ Universidad de Sucre
- ◯ Universidad del Cauca
- ◯ Universidad del Magdalena
- ◯ Universidad del Norte
- ◯ Universidad del Quindio
- ◯ Universidad del Rosario
- ◯ Universidad del Sinú Elias Bechara Zainúm (Cartagena)
- ◯ Universidad del Sinú Elias Bechara Zainúm (Monteria)
- ◯ Universidad del Tolima
- ◯ Universidad del Valle
- ◯ Universidad El Bosque
- ◯ Universidad Icesi
- ◯ Universidad Industrial de Santander
- ◯ Universidad Libre (Barranquilla)
- ◯ Universidad Libre (Cali)
- ◯ Universidad Metropolitana (Barranquilla)
- ◯ Universidad Militar Nueva Granada
- ◯ Universidad Nacional de Colombia
- ◯ Universidad Pedagógica y Tecnológica de Colombia
- ◯ Universidad Pontificia Bolivariana
- ◯ Universidad San Martín (Cali)
- ◯ Universidad San Martín (Sabaneta)
- ◯ Universidad Santiago de Cali
- ◯ Universidad Simón Bolívar
- ◯ Universidad Surcolombiana
- ◯ Universidad Tecnológica de Pereira
- ◯ Otro

Escriba el nombre de la universidad: ________________________________

¿Usted ha hecho algún tipo de práctica o entrenamiento fuera del país?

- ◯ Sí
- ◯ No

¿Durante cuántas semanas?	______________________________
¿Usted hará algún tipo de práctica o entrenamiento fuera del país?	◯ Sí<br>◯ No
¿Durante cuántas semanas?	______________________________
¿Cuáles de los siguientes cursos ha realizado usted hasta el momento? (Marque todas las respuestas que apliquen)	☐ Advanced Trauma Life Support (ATLS)<br>☐ Basic Life Support (BLS)<br>☐ Advanced Cardiac Life Support (ACLS)<br>☐ Pediatric Advanced Life Support (PALS)<br>☐ Neonatal Advanced Life Support (NALS)<br>☐ Minuto de Oro<br>☐ Clasificación, Evaluación y Tratamiento de Emergencias Pediátricas (CETEP)<br>☐ La Atención Integrada a las Enfermedades Prevalentes de la Infancia (AIEPI)<br>☐ Ninguno<br>☐ Otro
Escriba el nombre del curso:	______________________________
¿Usted ha realizado una rotación de medicina de urgencias?	◯ Sí, de 1 a 2 semanas<br>◯ Sí, de 3 a 4 semanas<br>◯ Sí, más de 4 semanas<br>◯ No<br>◯ No sé
Durante la rotación en medicina de urgencias, ¿Usted fue supervisado por un especialista de medicina de urgencias?	◯ Sí<br>◯ No<br>◯ No sé

Habilidades

1) ¿Cuántas veces ha colocado un dispositivo para asegurar la vía aérea (por ejemplo un tubo endotraqueal)? ____________________

2) ¿Cuántas veces ha realizado una descompresión de emergencia con aguja para un neumotorax a tensión? ____________________

3) ¿Cuántas veces ha realizado un taponamiento de heridas profundas con sangrado externo? ____________________

4) ¿Cuántas veces ha realizado inmovilización de la columna cervical? ____________________

Confianza

Se pide que mueva la barra deslizante a la posición que mejor indique la confianza que usted siente en cada situación de 0 (muy baja confianza) a 100 (muy alta confianza).

1) Manejar a un paciente con dificultad respiratoria: 0 — 100
(Place a mark on the scale above)

2) Manejar a un paciente con hipoxia crítica: 0 — 100
(Place a mark on the scale above)

3) Manejar a un paciente con un aumento severo del trabajo respiratorio: 0 — 100
(Place a mark on the scale above)

4) Manejar a un paciente con signos vitales inestables: 0 — 100
(Place a mark on the scale above)

5) Manejar a un paciente en choque: 0 — 100
(Place a mark on the scale above)

6) Resucitar activamente a un paciente inestable: 0 — 100
(Place a mark on the scale above)

7) Manejar a un paciente con trauma severo: 0 — 100
(Place a mark on the scale above)

8) Manejar a un paciente crítico después de un grave accidente de tránsito: 0 — 100
(Place a mark on the scale above)

9) Manejar a un paciente politraumatizado: 0 — 100
(Place a mark on the scale above)

10) Manejar a un paciente con una alteración severa del estado de conciencia: 0 — 100
(Place a mark on the scale above)

11) Manejar a un paciente en crisis convulsiva: 0 — 100
(Place a mark on the scale above)

12) Manejar a un paciente con agitación psicomotora: 0 — 100
(Place a mark on the scale above)

13) Manejar a un paciente crítico: 0 — 100
(Place a mark on the scale above)

.4) Colocar un dispositivo para asegurar la vía aérea (por ejemplo un tubo endotraqueal):

0 — 100

(Place a mark on the scale above)

.5) Realizar una descompresión de emergencia con aguja para un neumotorax a tensión:

0 — 100

(Place a mark on the scale above)

.6) Empaquetar una herida profunda para controlar un sangrado externo:

0 — 100

(Place a mark on the scale above)

.7) Realizar inmovilización de la columna cervical en un paciente con politraumatismo:

0 — 100

(Place a mark on the scale above)

Conocimiento Inicial

15 Preguntas de Conocimiento.

No habrá retroalimentación para estas preguntas y los resultados no serán compartidos a sus Facultades de Medicina. Por favor, no use recursos adicionales.

1) 1. Un hombre de 25 años ingresa a urgencias con severa alteración del estado de conciencia. Ingresa con ronquidos y respiración con gorgoteos. Usted nota que tiene las pupilas puntiformes. Sus signos vitales son FR 8 rpm, TA 90/60 mmHg, FC 112 lpm, SO2 84%. ¿Qué debería hacer PRIMERO?

- ◯ A. Realizar tracción mandibular
- ◯ B. Realizar un examen neurológico
- ◯ C. Solicitar un electrocardiograma
- ◯ D. Colocar un acceso venoso

2) 2. Un paciente ingresa después de un accidente en motocicleta. Él está gritando de dolor sin embargo obedece órdenes. A la auscultación murmullo vesicular conservado en ambos campos pulmonares. Su respiración no está comprometida, Su siguiente acción es:

- ◯ A. Exponer al paciente
- ◯ B. Tomar la presión arterial
- ◯ C. Calcular el Glasgow
- ◯ D. Solicitar una radiografía de tórax

3) 3. Un hombre anciano fue llevado al hospital por su familia después de un episodio de desmayo. El señor estuvo el día entero fuera de su casa. Al examinarlo, se encuentra pálido y su piel está fría y húmeda. No está respondiendo a las instrucciones verbales ni se está moviendo por sí solo. ¿Cuál acción debe realizar primero?

- ◯ A. Palpación del esternón.
- ◯ B. Verificar el pulso.
- ◯ C. Calcular la escala de coma de Glasgow.
- ◯ D. Obtener un acceso intravenoso

4) 4. Un hombre de 42 años ingresa después de ser apuñalado en el lado derecho de su tórax. Se queja de dolor en hemitórax derecho y (dificultad respiratoria) falta de aliento. Al examinar el paciente usted se nota que la tráquea esta desviada al lado contralateral y que hay ingurgitación yugular y a la auscultación tiene ruidos respiratorios disminuidos en hemitórax derecho. Su PA es 88/40 mmHg y FC es 100 lpm, con SO2 de 91%, ¿Cuál de los siguientes pasos es el más apropiado para su manejo?

- ◯ A. Administrar 1L de Líquidos Intravenosos
- ◯ B. Toracostomía con aguja
- ◯ C. Radiografía de tórax portátil
- ◯ D. Tubo de tórax

5) 5. Una mujer de 24 años, aproximadamente con 30 semanas de embarazo, ingresa después de ser atropellada por un carro que iba a alta velocidad. Ella está hablando y a la auscultación pulmonar se evidencia murmullo vesicular conservado en ambos campos pulmonares. Sus signos vitales son: FC 128 lpm, TA 85/45 mmHg, SO2 98%. ¿Cuál de los siguientes pasos es el mejor para la gestación?

- ◯ A. Administrar oxígeno suplementario
- ◯ B. Coloque a la paciente en decubito lateral derecho
- ◯ C. Coloque a la paciente en decubito lateral izquierdo
- ◯ D. Evaluar la fetocardia

6) 6. Un hombre de 52 años, ingresa por una laceración en la región medial del muslo derecho, posterior a un accidente en un sitio de construcción. Está sangrando profusamente a pesar de que los transeúntes taparon la laceración con gasa y mantuvieron presión significativa en el área por aproximadamente 30 minutos. El paciente se siente frío y está diaforético. ¿Qué usted debería hacer?

- ◯ A. Continuar con la presión local
- ◯ B. Hacer un torniquete distal a la laceración
- ◯ C. Insuflar un tensiómetro proximal a la herida
- ◯ D. Cubrir la laceración con gasas

7) 7. Un hombre de 56 años de contextura delgada, ingresa con severa falta de aliento. Él está sentado a un lado de la cama, incapaz de acostarse. Está taquipneico con sibilancias espiratorias e inspiratorias en ambos campos pulmonares. El paciente se encuentra alerta y es capaz de indicar y con la cabeza, pero no puede responder a las preguntas debido a su dificultad respiratoria. Sus signos vitales son: FR 30 rpm, TA 160/90 mmHg, FC 138 lpm, SO2 92%. ¿Cuál es su primera orden médica?

- ◯ A. Oxígeno suplementario
- ◯ B. 1L de Solución salina normal (SSN)
- ◯ C. Salbutamol
- ◯ D. Epinefrina

8) 8. Una niña de 7 años de edad, sin historia clínica previa, ingresa por dificultad respiratoria y sensación de opresión en la garganta posterior a una picadura de abeja; dice que su garganta está irritada y un poco apretada. Al examen físico tiene edema en los labios, sibilancias en ambos campos pulmonares y una erupción cutánea. ¿Cuál de los siguientes pasos debe hacer de inmediato?

- ◯ A. Oxígeno suplementario
- ◯ B. Epinefrina
- ◯ C. Aspirina
- ◯ D. Líquidos endovenosos

9) 9. Un hombre de 76 años ingresa al hospital con tos y dificultad respiratoria severa de un día de evolución. Sólo puede decir frases de 3 palabras, tiene una frecuencia respiratoria de 28 respiraciones por minuto, tiene una temperatura de 38.1°C, frecuencia cardiaca de 130 lpm y presión arterial 83/40 mmHg, y SO2 86%. ¿Cuál es la causa más probable de estos síntomas?

- ◯ A. Insuficiencia cardiac congestiva
- ◯ B. Neumonía
- ◯ C. Infarto Agudo del Miocardio
- ◯ D. Anemia

10) 10. Un niño de 6 años ingresa por fiebre y tos. Sus signos vitales son: Temperatura 40°C, FC 145 lpm, PA 80/40 mmHg, peso 15 Kg. Usted ordena un bolo de líquidos, la cantidad correcta de este, es:

- ◯ A. 200 ml
- ◯ B. 300 ml
- ◯ C. 400 ml
- ◯ D. 500 ml

11) 11. Una mujer de 89 años presenta con dificultad respiratoria que ha empeorado en los últimos días. Sus signos vitales son: Temperatura 36.2°C, FC 130 lpm, PA 88/65 mmHg, FR 30 rpm, SO2 88%. Al examen físico tiene sibilancias y crépitos en ambas bases pulmonares y edema con fóvea en ambos tobillos. ¿Cuál es el diagnóstico más probable?

- ◯ A. Choque cardiogénico
- ◯ B. Choque neurogénico
- ◯ C. Choque anafiláctico
- ◯ D. Choque séptico

.2) 12. Una mujer de 28 años sigue sangrando significativamente por su vagina después del parto de su primer hijo. ¿Cuál de las siguientes opciones sobre hemorragia posparto es cierta?

- ◯ A. Ocurre solamente en las primeras 48 horas posparto
- ◯ B. La causa más común es una laceración en el tracto genital bajo
- ◯ C. Después de resucitación con líquidos, la primera línea es oxitocina
- ◯ D. El masaje uterino es un tratamiento ineficaz

.3) 13. Un hombre de 19 años es traído para evaluación médica posterior a una caída. El paciente estaba patinando y cayó hacia atrás sobre el pavimento. Al examen físico tiene una hematoma en la parte posterior del cuero cabelludo; el paciente está acostado con los ojos cerrados pero los abre cuando lo llaman, responde a las preguntas pero parece confundido y se mueve las cuatro extremidades cuando se lo piden. ¿Cuál de los siguientes NO es parte de la escala de coma de Glasgow?

- ◯ A. Hematoma en la parte posterior del cuero cabelludo
- ◯ B. Abre los ojos cuando lo llama
- ◯ C. Parece confundido
- ◯ D. Se mueve las cuatro extremidades cuando se lo pide

.4) 14. Un hombre de 75 años es traído por su hija que está preocupada por el estado de confusión que tiene su padre. Según la hija, el paciente tiene antecedente de hipertensión arterial, dislipidemia, enfermedad arterial coronaria, diabetes insulino requirente y Enfermedad Renal Crónica. Recientemente le ajustaron los medicamentos, pero ella no puede recordar el nombre. Mientras examina al paciente, empieza a convulsionar. ¿Qué prueba ejecutaría primero?

- ◯ A. Glucometria
- ◯ B. Radiografía de tórax
- ◯ C. TAC de cráneo
- ◯ D. Electrocardiograma (ECG)

.5) 15. Un hombre que es habitante de la calle, de 46 años, presenta una crisis convulsiva. El personal de atención prehospitalaria encontró el paciente tirado en el suelo. Antes de llegar al hospital el paciente empieza a convulsionar. Durante el traslado, sus signos vitales son: FR 20 rpm, PA 90/50 mmHg, SO2 91%, su glucometría es 90 mg/dL. ¿Cuál es el medicamento que le suministrará primero?

- ◯ A. Antibióticos
- ◯ B. Benzodiacepina
- ◯ C. Dextrosa en agua destilada al 50%
- ◯ D. 1L de Solución salina normal (SSN)

Pensamientos Finales

1) ¿Qué más debemos saber o preguntar sobre este tema?
