# Supplementary material for: Confidence and knowledge in emergency management among medical students across Colombia: A role for the WHO basic emergency care course
Source: PLoS One. 2022 Jan 21;17(1):e0262282. doi: 10.1371/journal.pone.0262282 (PMC8782416; doi:10.1371/journal.pone.0262282)
Supplement: S2 File — (DOCX) [file pone.0262282.s002.docx]

**Defining complete knowledge and confidence scales**.

The single best answer knowledge questions were coded as binary variables as correct or incorrect. A complete knowledge score was defined as 14 or more questions answered (Supplemental Table 1). Those with less than 14 questions answered were removed from analysis. Missing items were coded as incorrect for those with 14 or more questions answered.

A complete confidence scale was defined as >10 items answered (Supplemental Table 2). Those answering </= 10 items were removed from analysis. The visual analog scale assessing confidence was initially positioned at 50%. Thus, an item where participant who felt 50% confident and therefore, did not move the slider would record as missing. For those completing >10 confidence scales, missing values were set to 50%. .

| Number of Knowledge Questions completed | Frequency (%)  N=714 | Frequency (%) of last question answered for each group. |
| --- | --- | --- |
| 0 | 182 (25.5) | 0 (0) |
| 1 | 40 (5.6) | 0 (0) |
| 10 | 1 (0.1) | 0 (0) |
| **14** | **96 (13.5)** | **93 (96.8)** |
| **15** | **395 (55.3)** | **395 (100)** |

Supplemental Table 1: Knowledge section completion of those consenting to participate with completion of the final question. Rows bolded were included in final analysis.

| **Number of Confidence Items completed** | **Frequency (%)**  **N=714** | **Frequency (%) of last question answered for each row.** |
| --- | --- | --- |
| 0 | 144 (20.17) | 0 (0) |
| 1 | 4 (0.6) | 0 (0) |
| 2 | 1 (0.1) | 0 (0) |
| 4 | 2 (0.3) | 0 (0) |
| 5 | 1 (0.1) | 1 (100) |
| 6 | 1 (0.1) | 0 (0) |
| 7 | 2 (0.3) | 1 (50) |
| 8 | 2 (0.3) | 0 (0) |
| 9 | 5 (0.7) | 2 (40) |
| 10 | 6 (0.8) | 3 (50) |
| **11** | **19 (2.7)** | **16 (84.2)** |
| **12** | **41 (5.7)** | **38 (92.7)** |
| **13** | **486 68.1)** | **100 (100)** |

Supplemental Table 2: Confidence section completion of those consenting to participate. Within each row, completion of the final question is counted. Rows bolded were included in final analysis.

| **Training Course** | **# (%)** | **Mean % Knowledge  Score (95% CI)** | **P value** | **Mean Confidence  Score (95% CI)** | **P Value** |
| --- | --- | --- | --- | --- | --- |
| ATLS* | 171 (36.5) | 58.2 (54.6-61.9) | 0.253 | 62.9 (60.7-65.1) | 0.0014 |
| BLS* | 291 (62.2) | 65.1 (62.6-67.6) | <0.001 | 63.5 (61.8-65.2) | <0.001 |
| ACLS* | 242 (51.7) | 67.5 (65.0-70.1) | <0.001 | 63.4 (61.8-65.4) | <0.001 |
| PALS* | 140 (30.0) | 67.8 (64.5-71.2) | <0.001 | 62.8 (60.3-65.3) | 0.0073 |
| NALS* | 113 (24.2) | 67.8 (64.0-71.6) | <0.001 | 62.8 (60.2-65.5) | 0.0194 |
| Minuto de Oro | 70 (15.0) | 70.2 (65.9-74.5) | 0.001 | 59.4 (56.0-62.8) | 0.8893 |
| CETEP* | 6 (1.3) | 72.2 (57.3-87.2) | 0.193 | 53.2 (38.6-67.8) | 0.3439 |
| AIEPI* | 205 (43.8) | 65.2 (62.2-68.3) | <0.001 | 61.5 (59.5-63.4) | 0.0372 |
| Other | 27 (5.8) | 58.1 (48.8-67.2) | 0.6723 | 54.9 (48.4-61.5) | 0.132 |
| *Missing* | *3 (0.6)* |  |  |  |  |

Supplemental Table 3: Previous emergency care course completions and associated mean percent knowledge score and mean confidence score among graduating medical students across Colombia compared to the overall knowledge score (59.9% 95% CI 57.8-62.0%) or the overall confidence score for the entire cohort (59.6 mm 95% CI 58.1-61.2 mm).

*ATLS: Advanced Trauma Life Support

*BLS: Basic Life Support

*ACLS: Advanced Cardiac Life Support

*PALS: Pediatric Advanced Life Support

*NALS: Neonatal Advanced Life Support

*CETEP: Centro Técnico de Educacíon Profesional

*AIEPI: Atención Integral a las Enfermedades Prevalentes en la Infancia

|  | **Removed**  **N=246** | **In Study Sample**  **N=468** | **P Value**  **Chi^2^** |
| --- | --- | --- | --- |
| **Age** | | | |
| 19-24 | 87 (35.4) | 326 (69.7) | 0.2 |
| 25-44 | 45 (18.3) | 130 (27.8) |  |
| *Missing* | *114 (46.3)* | *12 (2.6)* |  |
| **Sex** | | | |
| Male | 41 (16.7) | 99 (21.2) |  |
| Female | 174 (70.7) | 292 (62.4) | 0.2 |
| *Missing* | *106 (43.1)* | *2 (0.4)* |  |
| **Region:** | | | |
| Pacífico | 15 (6.1) | 31 (6.6) |  |
| Caribe | 30 (12.2) | 86 (18.4) |  |
| Andes | 86 (35.0) | 311 (66.5) |  |
| Orinoquía | 6 (2.4) | 38 (8.1) | 0.1 |
| *Missing* | *109 (44.3)* | *2 (0.4)* |  |
| **Number of Training Courses** | | | |
| 0 | 33 (13.4) | 83 (17.7) |  |
| 1 to 3 | 52 (22) | 212 (45.3) |  |
| 4 to 7 | 52 (21.1) | 170 (36.3) | 0.17 |
| *Missing* | *109 (44.3)* | *3 (0.6)* |  |
| **Number of times performing a task:** | | | |
| *Securing an airway* | | | |
| 0 | 34 (13.8) | 131 (28.0) |  |
| 1 to 5 | 60 (24.4) | 239 (51.1) |  |
| >5 | 28 (11.4) | 96 (20.5) | 0.2 |
| *Missing* | *124 (50.4)* | *2 (0.4)* |  |
| *Chest Tube Placement* | | | |
| 0 | 89 (36.2) | 356 (76.1) |  |
| 1 to 5 | 27 (11.0) | 99 (21.2) |  |
| >5 | 6 (2.4) | 10 (2.1) | 0.3 |
| *Missing* | *124 (50.4)* | *3 (0.6)* |  |
| *External hemorrhage control :* | | | |
| 0 | 83 (33.7) | 227 (48.5) |  |
| 1 to 5 | 53 (21.5) | 217 (46.4) |  |
| >5 | 4 (1.6) | 23 (4.9) | 0.2 |
| *Missing* | *106 (43.1)* | *1 (0.2)* |  |

Supplemental Table 4: Characteristics of the study sample as compared to those removed via case deletion via Chi2 analysis.

| **Geographic**  **Region** | **National Distribution (%)** | **Study Sample Distribution (%)** | **P Value (Chi^2^)** |
| --- | --- | --- | --- |
| Pacífico | 8.2 | 6.6 |  |
| Caribe | 29.3 | 18.4 |  |
| Andes | 61.2 | 66.5 |  |
| Orinoquía | 1.3 | 8.1 | 0.005 |

Supplemental Table 5: Geographic distribution of the national medical student population versus the study sample distribution.
